# New Methodologies to Study DNA Repair Processes in Space and Time Within Living Cells

**DOI:** 10.3389/fcell.2021.730998

**Published:** 2021-09-13

**Authors:** Siham Zentout, Rebecca Smith, Marine Jacquier, Sébastien Huet

**Affiliations:** ^1^Univ Rennes, CNRS, IGDR (Institut de Génétique et Développement de Rennes)-UMR 6290, BIOSIT-UMS 3480, Rennes, France; ^2^Institut Universitaire de France, Paris, France

**Keywords:** DNA damage, live cell imaging, fluorescence fluctuation analysis, spatio-temporal analysis, kinetic modeling

## Abstract

DNA repair requires a coordinated effort from an array of factors that play different roles in the DNA damage response from recognizing and signaling the presence of a break, creating a repair competent environment, and physically repairing the lesion. Due to the rapid nature of many of these events, live-cell microscopy has become an invaluable method to study this process. In this review we outline commonly used tools to induce DNA damage under the microscope and discuss spatio-temporal analysis tools that can bring added information regarding protein dynamics at sites of damage. In particular, we show how to go beyond the classical analysis of protein recruitment curves to be able to assess the dynamic association of the repair factors with the DNA lesions as well as the target-search strategies used to efficiently find these lesions. Finally, we discuss how the use of mathematical models, combined with experimental evidence, can be used to better interpret the complex dynamics of repair proteins at DNA lesions.

## DNA Repair: A Multistep Process Coordinated in Space and Time

**FIGURE 1:**
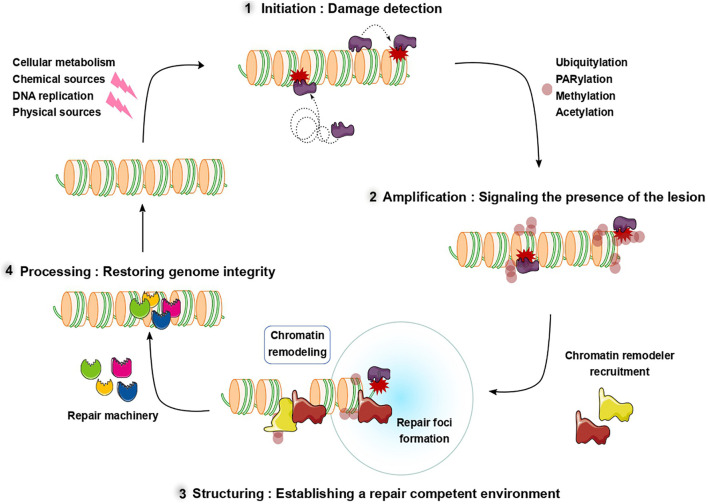
Schematic overview of the multiple steps of the DNA repair process. After damage induction, sensor proteins recognize and recruit to DNA lesions (1) initiating the DNA damage response. In the second phase (2), post-translational modifications signal the presence of the damage and promote the recruitment of downstream repair factors including chromatin remodelers. These downstream factors restructure chromatin to establish a repair competent environment (3) allowing efficient repair. After the DNA lesions are repaired, chromatin is restored to a pre-damaged conformation (4).

Throughout the lifetime of a cell, its genome is continuously challenged by a variety of stresses which can originate from the cell itself, including metabolic byproducts, or from external sources such as environmental mutagens or radiations (reviewed in Chatterjee and Walker, 2017). These genomic stresses can result in a variety of lesions ranging from base modifications to single- and double-strand breaks (SSBs and DSBs) (Carusillo and Mussolino, 2020). To detect and restore the genomic integrity, cells use highly sophisticated mechanisms, often gathered within the generic term of DNA damage response (DDR). To preserve the genome and avoid accumulation of mutations deleterious for the cell or promoting tumorigenesis, the DDR must achieve two objectives: (i) it has to be highly efficient, meaning that the lesions need to be detected both rapidly and exhaustively, and (ii) it must be accurate, restoring genomic integrity not only at the DNA level, but also at higher scales of the genome organization such as the chromatin folding or the epigenetic marks (Dabin et al., 2016). To fulfill these two criteria, the DDR is organized in a multistep process which will be described in the following (Figure 1).

### The Initiation Step: Navigating the Crowded Nucleus to Efficiently Detect DNA Lesions

In each human nucleus, about two meters of DNA is wrapped around histone proteins to form a chromatin fiber which itself needs to be folded to fit within a nucleus with a diameter of about 10 μm (Pombo and Dillon, 2015; Ou et al., 2017). It is this dense and complex structure that needs to be constantly scanned by the DNA repair machinery to detect the presence of DNA lesions. This detection is performed by proteins that can sense specific DNA lesions, such as the DNA-glycosylase OGG1 which detects the oxidized form of guanine (D’Augustin et al., 2020) or the Ku complex which binds to DNA ends consecutive to DSBs (Ma et al., 2020). Nevertheless, each sensor faces a paradox: it needs to scan the DNA quickly, to allow a rapid detection of rare lesions, but also needs to be highly specific, which requires a careful and potentially lengthy inspection of the DNA to avoid missing a lesion or initiating illegitimate repair. The strategies developed by the sensors of DNA lesions to resolve this speed/specificity paradox remains the subject of intense research. A common trait shared by many of these sensors is that they explore the nuclear volume by alternating phases of 3D diffusion within the nucleoplasm with transient aspecific binding onto the DNA, which may itself involve short diffusive scans along the double helix (Woringer and Darzacq, 2018). This complex dynamic, often referred as facilitated diffusion, is strongly impacted by the local architecture displayed by the chromatin. It seems obvious that compacted chromatin domains may partially hinder lesion detection but more complex effects of the spatial topology of the chromatin/nucleoplasm interface have also been reported (Baum et al., 2014). Indeed, theoretical and experimental work predict that the smoothness of this interface may impact how exhaustive the search process will be (Condamin et al., 2008; Bancaud et al., 2009).

### The Amplification Step: Signaling the Presence of the Lesions

Because the search step might be tedious, once a lesion has been detected, its localization needs to be clearly highlighted to facilitate further repair steps. This highlighting is ensured by multiple signaling pathways that mark the chromatin with specific post-translational modifications (PTMs). A typical example is poly(ADP-ribosyl)ation (PARylation) signaling, which is essential at early steps of the SSB repair and also important for resolving other types of damage. Upon binding to a DNA lesion, the poly(ADP-ribose) polymerase PARP1 will catalyze negatively charged PAR chains on itself and on surrounding proteins in particular histones located nearby the lesion (Kamaletdinova et al., 2019). These PAR chains are recognized by several effector proteins, which promotes their accumulation at the sites of damage. Similar processes occur at DSBs, where the initial complexes detecting these lesions contain the kinases ATM, ATR, or DNA-PKcs, which are responsible for marking the nearby chromatin, among many other regulatory functions (Her and Bunting, 2018; Wright et al., 2018). Therefore, this signaling step, combined to specific protein/protein interactions, amplifies the initial trigger emanating from the sensor proteins. This allows for the local concentration of repair actors, often leading to the formation of so-called repair foci, which is a classical strategy used by the cell to accelerate biochemical reactions.

### The Structuring Step: Establishing a Repair Competent Environment

Signaling the presence of the DNA lesion not only promotes the recruitment of later repair actors, but is also crucial to establish an environment favorable to efficient repair (Yasuhara and Zou, 2021). In particular, this involves complex chromatin restructuring processes aimed at facilitating the access to DNA lesions as well as their processing (Smith et al., 2019). These chromatin remodeling processes are controlled by several post-translational modifications targeting histones as well as chromatin remodelers and histone chaperones (Piquet et al., 2018; Rother et al., 2020). This structuring step not only involves changes in the chromatin architecture, but it likely also promotes the establishment of properly organized repair foci. 53BP1 (Mirman and de Lange, 2020) is recruited to DBSs in response to a combination of signaling cues involving histone ubiquitination and methylation and contributes to the formation of repair foci by establishing a recruitment platform for multiple other repair factors (Mirza-Aghazadeh-Attari et al., 2019; Lou et al., 2020). More recently, 53BP1 was also shown to promote liquid-liquid unmixing, a process that could help accumulate factors within repair foci without the need for specific protein/protein interactions (Kilic et al., 2019; Ghodke et al., 2021). Importantly, these 53BP1 foci were also proposed to locally hold the chromatin architecture, to keep it in a configuration favorable for repair (Ochs et al., 2019). Therefore, altogether, the different actors involved in this structuring step, although not directly participating to the resolution of the DNA lesion, can improve the efficiency of the repair and also potentially dictate the pathway that will be chosen for restoring the genome (Xu and Xu, 2020). Indeed, while the early chromatin “breathing” triggered by the joint activities of CHD7 and HDAC1/2 promotes DSB repair by non-homologous end-joining (NHEJ) (Rother et al., 2020), chromatin remodeling via CHD4 rather seems to favor DSB repair by homologous recombination (Qi et al., 2016; Smith et al., 2018).

### The Processing Step: Restoring the Genome Integrity

All the steps mentioned so far were important to initiate the restoration of the genome integrity but none of them directly participate in the processing of the DNA lesions. This key step is ensured by sets of actors that each fulfill a specific function. For example, in the context of base excision repair, the damaged base is first excised by a dedicated glycosylase (D’Augustin et al., 2020). This leaves an abasic site that is itself processed by the endonuclease APE1, generating a single-strand break that is then resolved by the combined action of specific DNA polymerases and ligases (Abbotts and Wilson, 2017). Obviously, the choice of the actors involved in lesion resolution depends on the initial detection event but, as described in the previous section, is also controlled by later steps of the DDR that integrate several sources of information: the type of lesion, the local chromatin landscape, as well as the cell-cycle stage (Hustedt and Durocher, 2017; Her and Bunting, 2018; Schep et al., 2021). Importantly, restoring genome integrity is not restricted to the recovery of the original DNA sequence, it also involves the reestablishment of the chromatin landscape. The activity of several histone chaperones is needed at late stage of the repair process (Chen et al., 2008). These chaperones probably participate in depositing specific histones such as the H3.3 variant, a process that is needed to shut down DNA damage signaling and allow transcription recovery at the damage locus (Kim and Haber, 2009; Adam et al., 2013).

This brief introduction regarding the key steps of the DDR demonstrates that this process is a spatio-temporal orchestra involving a large number of instruments. The studies performed over the last decades have allowed the specific function of many of the repair factors to be uncovered but the current challenge in the field is now to identify the bandmasters able to coordinate all these factors to get them playing in tune and allow efficient repair. Addressing this difficult question relies in particular on the use of quantitative tools able to assess at high spatio-temporal resolution the dynamics of the different repair factors at the sites of damage, but also within the rest of the nucleus. In the following, we will review the tools deriving from fluorescence imaging that are currently available to monitor in living cells the multiple steps of the DDR.

## Tools to Assess Recruitment Kinetics at Sites of Damage

### Expressing the Needs: The Right Damage in the Right Place at the Right Time

As described in the previous section, there is a need for a better description of the spatio-temporal dynamics of the repair actors within the cell nucleus after DNA damage induction in addition to the biochemical characterization of the repair machinery. Live-cell fluorescence microscopy is the method of choice to address this question. Using classical single-beam scanning or spinning-disk confocal imaging, one can follow protein dynamics at timescales ranging from tens of milliseconds to hours, with a spatial resolution of few hundreds of nanometers within the 3D space of the nucleus of individual cells (Aleksandrov et al., 2018). Higher spatial resolutions can be achieved by using methods such as stimulated emission depletion or structured illumination microscopy, although this is usually at the expense of the speed of acquisition (Ochs et al., 2019). Ultimately, single-molecule imaging methods allow the behavior of individual repair proteins to be monitored (see below section “Single-Molecule Approaches to Assess Protein Turnover at Sites of Damage” for more details). They remain, however, difficult to use for non-experts and therefore have not yet been applied extensively in the DNA repair field despite having the potential to provide highly valuable information about protein dynamics (Miné-Hattab et al., 2021).

While all the fluorescence microscopy methods mentioned above have been used to study the dynamics of multiple intracellular processes, a specificity of the DNA repair field is that these imaging techniques need to be combined with a way to inflict DNA lesions to be able to follow the cellular response. Ideally, the DNA damaging method should allow a single type of lesion to be induced at a predefined location in the genome and at a time point that can be precisely estimated. It is only under such circumstances that it will be possible to precisely assess the sequence of events associated with the repair of a given type of lesion in the context of a particular chromatin landscape. Unfortunately, to date, such an ideal DNA damaging method does not exist. In the following we will review the methods that are currently available to induce DNA damage and to follow the DNA damage response in living cells using microscopy. We will show how each of these methods only fulfills some of the three criteria mentioned above, making them more or less suitable depending on the question of interest.

### Genotoxic Agents, Nucleases, Irradiation: Different Ways to Induce DNA Damage to Answer Different Questions

Three main approaches are currently in use to induce DNA damage in the context of live cell imaging: genotoxic drugs, endonuclease targeting and irradiation using various sources (Table 1). Genotoxic agents have been used for many years to induce DNA lesions, with the advantage that some of them are used in the clinic as anticancer agents. These agents display two modes of action. They can either directly alter the DNA or inhibit the activity of some cellular factors, ultimately leading to DNA damage. A well-known example of the first category of genotoxic agent is cisplatin, which induces intra- or inter-strand crosslinks (Cohen and Lippard, 2001; Hu et al., 2016). Inhibitors of the topoisomerases are a prominent family of molecules within the second category of genotoxic agents (Xu and Her, 2015). These different types of molecules have been used extensively within the DNA repair field. However, as they tend to induce multiple types of damage relatively evenly within the genome and since the time of damage induction cannot be precisely estimated, these genotoxic agents are often not compatible with a precise spatio-temporal characterization of the DNA damage response.

**TABLE 1:** Comparison between the different methods used to induce DNA lesions.

Tools	Induction of a single type of DNA lesion	Ability to choose the genomic location	Synchronization of damage induction	Characterization of early steps of DNA repair
Genotoxic drugs	+	–	–	–
Endonuclease targeting	+++	+++	+	+
Microirradiation	–	+	+++	+++

To be able to induce a specific type of lesion at a given locus, several approaches have been developed over the last years based on DNA endonucleases. The expression of I-SceI in cells whose genome integrates the 18-bp recognition site of this nuclease (Rouet et al., 1994) or the use of a construct associating the nuclease domain of the *Fok*I enzymes with the Lac repressor/Lac operator assay (Shanbhag et al., 2010) allows for the induction of DSBs at one or few known locations in the genome. The restriction enzyme *Asi*SI, which recognizes about 150 endogenous sequences along the genome, can also generate multiple DSBs within a given nucleus (Iacovoni et al., 2010). More recently, programmable endonucleases such as Zinc-finger nucleases or CRISPR-Cas9 have been used to induce either single or multiple DSBs at different genomic loci (Morton et al., 2006; Wang et al., 2019; Emmanouilidis et al., 2021, 9). Interestingly, some of these enzymes have been mutated to switch from a nuclease to a nickase activity, allowing to generate SSBs (Davis and Maizels, 2014). This strategy is nevertheless inherently limited to the study of DNA breaks. Another limitation of this approach is the poor resolution regarding the timing of damage induction which precludes a precise analysis of the sequence of events composing the DDR. To circumvent this limitation, inducible systems have been developed by fusing the nucleases to nuclear receptors to allow the relocalization of these fusion proteins upon addition of the receptor agonist (Soutoglou et al., 2007; Caron et al., 2012, 2015). More recently, a strategy based on light-inducible uncaging of the guide RNA has also been proposed to trigger damage induction with Cas9 at a specific timepoint (Liu et al., 2020). With these inducible methods, it is possible to reach a precision of a few minutes in terms of the timing of damage induction. While sufficient to analyze repair processes displaying characteristic timescales of tens of minutes or hours, this time resolution is not suitable to monitor the early fast steps of the DDR. The last limitation of the nuclease strategy is the risk of recurrent damage since these enzymes have the potential to reinitiate cleavage as soon as the break is resolved. These breaks may be recognized as unrepairable, leading to the activation of specific pathways (Oza et al., 2009). To limit this problem, some authors have proposed the use of auxin-inducible degron to degrade the nuclease within a timeframe of approximately half an hour and therefore stop damage induction (Aymard et al., 2014).

The third method to induce DNA lesions under the microscope is based on irradiation. This approach allows DNA lesions to be induced locally within the nucleus, with the extent of the damage area depending on the size of the irradiation beam. With this approach, the precise timing of irradiation is known, making this DNA damaging approach particularly suitable for characterizing the initial steps of the DDR (Gassman and Wilson, 2015; Aleksandrov et al., 2018). Nevertheless, the main drawback of irradiation is that it usually does not lead to the formation of a single type of DNA lesion but rather creates a mixture of damage which are clustered within the irradiation area (Schipler and Iliakis, 2013). The induction of such complex array of DNA damage types are not only problematic for the study of specific repair pathways, potentially leading to seemingly contradictory results depending on the irradiation method, but also represent a major challenge for the cell that may experience difficulties to efficiently resolve these genomic alterations (Asaithamby et al., 2011). In the following, we will briefly review the different irradiation methods that are currently available and describe the different types of DNA lesions that they induce.

### Various Modalities of Irradiations for Different Types of DNA Damage

DNA damage induced by irradiation gathers a large number of approaches that differs not only from the type of irradiation sources but also the design of the irradiation scheme in space and time as well as the potential use of chemical sensitizers. Ionizing radiations such as γ-rays, X-rays, or ion beams have been used extensively to generate DNA lesions around which form the so-called ionizing radiation-induced foci (IRIF), where different repair factors accumulate (van Veelen et al., 2005; Jakob et al., 2009; Costes et al., 2010). Depending on the ionizing radiation, the type of lesions that are created can be mainly SSBs and DSBs but more complex types of damage are also observed (Ward, 1988; Datta et al., 2005). One motivation for the use of these ionizing radiation is that they are similar to those used in anticancer radiotherapies and therefore, the analysis of the cellular response improves our understanding of the molecular mechanisms underlying this therapeutic strategy (Mohamad et al., 2017). Nevertheless, the access to some of these radiation sources might be limited. Moreover, directly coupling these sources with fluorescence imaging setups, which are required to monitor the DDR in living cells from its early stages, remains challenging (Hable et al., 2012; Jakob et al., 2020).

The most common sources of irradiation used in the context of the study of the DDR by live cell imaging are the lasers that are either already present in the common set of lines used to excite fluorescence, or that can easily be coupled to the microscope (Holton et al., 2017). Irradiation lasers are divided in two main categories, continuous and pulsed lasers. Continuous 405 nm lasers are available on most confocal setups and therefore represent a widely used method to induce DNA lesions, provided that the cells have been pretreated with sensitizing agents such as the DNA intercalating agent Hoechst 33342 or the nucleotide analog BrdU (Singh et al., 2004; Suzuki et al., 2010; Vítor et al., 2020). Pulsed lasers induce DNA lesions without the need for such photosensitizers. They are often in the near UV (∼350 nm) or infrared ranges (∼800 nm), sparing the visible window for fluorescence imaging. Shorter UV wavelengths are also available although they require the use of specific lenses (Kong et al., 2009; Gassman and Wilson, 2015). Besides their emission wavelengths, these lasers also differ significantly by the duration of the pulses, which ranges from about 150 femtoseconds to few nanoseconds, as well as the pulse rates, which cover six orders of magnitudes. Irradiation with these different laser sources generate multiple types of DNA lesions: base oxidation, crosslinks, SSBs, or DSBs. Although the relative abundance of these types of damage within the irradiated area differs depending on the laser, it is probably difficult to find irradiation conditions that induce only a single type of lesion (Dinant et al., 2007; Kong et al., 2009). With regards to pulsed lasers, nanosurgery data (Colombelli et al., 2007) suggests that shorter pulses induce more local and potentially cleaner cuts, but it remains unclear whether this also holds true for irradiation aiming at inducing DNA lesions.

An interesting new approach is the combination of laser irradiation with targetable photosensitizers. These photosensitizers can be genetically encoded fluorophores such as the Killer-red that tends to generate reactive oxygen species (ROS) upon illumination (Lan et al., 2014), or fluorogen-activating proteins (FAP) that bind a photosensitizer ligand (He et al., 2016). These combined methods might be more specific than simple laser irradiation to induce a particular type of damage such as base oxidation. Moreover, fusing Killer-red or the FAP to domains that localize to specific genomic loci would allow DNA lesions to be introduced within predefined chromatin regions, something that is not easily manageable with simple laser irradiation. Such an example of this targetable DNA damage approach was recently described in a report by Fouquerel et al., in which FAP was fused to TRF1 to allow inducing base oxidation specifically at telomeres (Fouquerel et al., 2019).

Despite the limitations described above, laser irradiation currently remains the method of choice to assess the sequence of processes occurring during the DDR with a well-defined time origin corresponding to the irradiation event. In particular, multicolor live-cell imaging allows easy monitoring of recruitment kinetics of several fluorescently tagged proteins in parallel to the sites of damage (Garbrecht et al., 2018). While the crosstalk between the spectrum of the different fluorophores makes it difficult to assess simultaneously more than three repair factors (Tie and Lu, 2020), a quantitative analysis of the recruitment kinetics of proteins expressed in separated cells still allows for a detailed picture of the complex choreography taking place at DNA lesions to be drawn. In the following section, we will describe tools currently available for such quantification as well as the parameters that can be extracted from this analysis.

### Extracting Accurate and Quantitative Information From the Recruitment Data

Current imaging setups allow the accumulation and release of fluorescently labeled repair factors from sites of laser irradiation to be recorded at timescales ranging from tens of milliseconds to hours (Aleksandrov et al., 2018). Classically, a tagged version of the protein of interest is expressed in living cells by transient transfection or by establishing stably expressing clones. However, this results in protein overexpression which can create an imbalance between the different actors of the DDR, potentially leading to artifacts. To overcome this problem, the tagging of the endogenous protein can be achieved by CRISPR/Cas9 based genome editing (Stewart-Ornstein and Lahav, 2016) or via the use of fluorescently labeled nanobodies raised against the repair proteins of interest (Buchfellner et al., 2016; de Beer and Giepmans, 2020). Importantly, the association between the nanobody and its target must be tight enough to ensure that the dynamics of the fluorescence distribution adequately represents the one of the repair protein, but it should also have no impact the function of this protein (Buchfellner et al., 2016). Image sequences of cells expressing the fluorescently tagged repair factors can then be recorded by timelapse imaging after laser irradiation. To be able to precisely assess the recruitment kinetics of these factors at DNA lesions, the first step consists in the quantification of the fluorescence signal within the irradiated area. Manually delineating such region of interest (ROI) on the image sequences can easily be performed using any image-processing tool. However, this manual approach may introduce some user-bias and may also be tedious if the ROI needs to be updated due for example to cell movement during the timecourse (Lebeaupin et al., 2017). Alternatively, automatic segmentation tools can be used to identify the irradiation ROI based on the spatial distribution of the repair protein of interest. The drawback of such approach is that the segmented area may differ from one repair protein to another and may also evolve along with the recruitment kinetic since strong protein accumulation will lead to the segmentation of larger ROIs than for fainter ones. A more appropriate strategy is to include an additional marker that is independent from the repair factors but identifies the irradiated region. This can be achieved by the use of photoactivatable proteins, whose fluorescence is activated by the laser used for irradiation, fused to core histones (Sellou et al., 2016). The irradiated area is then highlighted with good signal-to-noise ratio, allowing an easy automatic segmentation that does not depend on the repair protein of interest. Fluorescent proteins such as PA-GFP (photoactivatable GFP) or PA-TagRFP can be easily converted with continuous 405 nm or pulsed near-infrared lasers (Testa et al., 2008; Lebeaupin et al., 2017). When using other lasers to induce damage, one possibility is to combine them with a 405 nm illumination. Indeed, provided that the cells are not presensitized, the level of 405 nm illumination needed to induce photoactivation is too low to induce significant DNA lesions (Lebeaupin et al., 2017).

Based on the segmentation of the irradiation area as well as the one of a whole nucleus, it is then possible to estimate the overaccumulation of repair proteins at sites of damage relative to the rest of the nucleus and to monitor the temporal evolution of this overaccumulation. Several classical parameters can then be readily extracted from these recruitment curves such as, for example, the time and amplitude of the recruitment peak or the time needed for dissipating half of the peak accumulation (Mistrik et al., 2016; Prokhorova et al., 2021). Alternatively, when focusing specifically on the accumulation phase of the curve, phenomenological models such as first or second order response to a step change can be used to extract characteristic rising times (Bekker-Jensen et al., 2005). These different parameters are useful to compare the relative kinetics of different repair proteins and cluster them based on their dynamic behavior at DNA lesions (Kochan et al., 2017; Garbrecht et al., 2018) but they do not bring much information regarding the molecular mechanisms underlying the accumulation and release of the repair factors. To go one step further, the individual recruitment curves can be fitted with analytical models assuming different scenarios including one or multiple step reactions for the accumulation and the dissipation phases as well as characteristic residency times at DNA lesion. Recently, the Stoynov team analyzed the recruitment kinetics of 70 proteins using such models (Aleksandrov et al., 2018). By clustering the repair proteins based on their characteristic accumulation and dissipation times, they were able to propose some coordination mechanisms between factors in charge of repairing different DNA lesions at sites of irradiation containing multiple types of damages.

A simplification common to all these analytical models is to consider that the diffusion of the repair proteins within the nuclear volume is instantaneous and therefore, that the recruitment kinetics are governed solely by reactions rates associated with accumulation and dissipation at the DNA lesions. Nevertheless, it has been shown that multiple chromatin-interacting proteins display diffusion-limited dynamics in the nucleus (Beaudouin et al., 2006), indicating that protein displacement within the nuclear space needs to be taken into account in addition to the binding and unbinding events onto the chromatin. Unfortunately, differential equations that are derived from models that take both the reaction and diffusion components into account usually cannot be solved analytically, thus precluding a simple fit of the experimental recruitment data with a predefined mathematical expression. Therefore, more elaborated approaches involving the fitting of the data with numerically solved reaction-diffusion models need to be implemented. A clear demonstration of the interest to develop such strategies has been recently highlighted by work from the Luger lab (Mahadevan et al., 2019; Bowerman et al., 2021). Using a Monte-Carlo based model that assumes the repair factors can either diffuse by pure Brownian motion or bind to the DNA lesions, Mahadevan et al. were able to simulate their accumulation kinetics at DNA lesions and adjust these simulations to the experimental data. They show that the nuclear shape has a strong influence on the recruitment curves, highlighting the fact that space matters for repair factors that explore the nucleus searching for DNA lesions. This work opens the way for more complex numerical models describing not only the rising of the recruitment curves but also their decline (see section “The Need for a Quantitative Model to Integrate the Data Obtained From Different Tools” below).

Refined models of the reaction-diffusion processes occurring at sites of damage necessarily come with more unknown parameters. Getting a precise estimation of these parameters requires an increase in data sample size to better catch the cell-to-cell variability. This is achievable by combining regular irradiation patterns designed to hit tens of cells within the microscope field of view simultaneously (Mistrik et al., 2016), with automated analysis pipelines (Oeck et al., 2019). But besides acquiring more data, a better description of the behavior of the repair factors at sites of damage also requires to extend beyond the analysis of recruitment curves. In the next section, we will show how the characterization of the turnover of the repair factors at sites of damage can bring crucial information to improve our understanding of the mechanisms regulating the successive steps of the DDR.

## Going Beyond Recruitment Kinetics: The Tools to Assess Protein Turnover at Sites of DNA Damage

### Reasons for Investigating the Turnover of Repair Proteins at Sites of Damage

The development of fluorescence microscopy methods over the last decades have shown that, while the analysis of the steady-state localization of proteins in fixed or live tissues brings valuable information about the function of these proteins, analyzing their dynamics is also essential to understand how this localization is regulated and therefore better describe the molecular mechanisms underlying these functions (Lippincott-Schwartz et al., 2001, 2003). The DNA repair field appears relatively unexplored regarding these questions of protein dynamics compared to related topics such as transcription control (Kimura et al., 2002; Mueller et al., 2008, 2010). Indeed, while the recruitment kinetics of repair proteins at DNA lesions have been studied extensively as described in the previous section, only a limited number of studies addressed the question of the local turnover of these factors within the damaged area.

**FIGURE 2:**
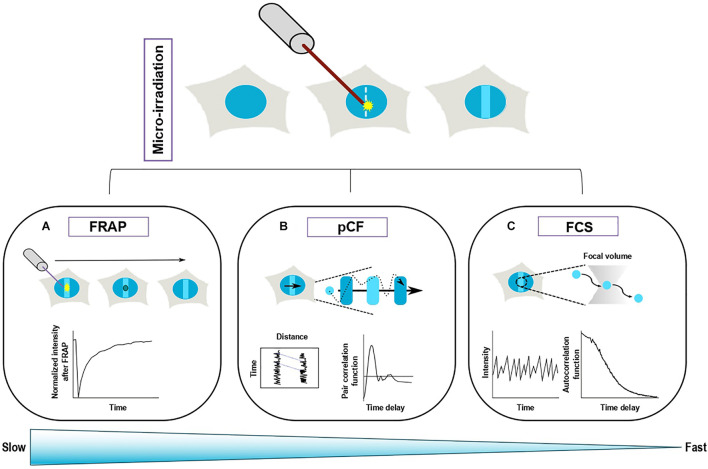
Complementary fluorescence-based methods to study the turnover of repair proteins at sites of DNA damage. Top panel: Recruitment of a fluorescently tagged protein at sites of DNA damage induced by laser irradiation. Bottom panel: Three different methods allow to monitor protein turnover at sites of damage. **(A)** Fluorescence recovery after photobleaching (FRAP) is based on the photobleaching of a sub-region (solid circle) of the area of DNA damage. The fluorescence recovery within the bleached area gives access to protein turnover at DNA lesions. **(B)** Pair correlation function (pCF) assesses the movements of the proteins within a line-scan across the damage region. The fluorescence signals from two different pixels along the acquisition line are cross-correlated, which allows to estimate the characteristic transit time between these two pixels. **(C)** Fluorescence Correlation spectroscopy (FCS) collects the fluctuations arising from the movement of fluorescently tagged proteins in and out of a confocal volume located within the DNA damage region. The characteristics of the protein turnover at sites of damage are derived from the analysis of the autocorrelation of the fluorescence fluctuations. FRAP is more appropriate to assess slow turonver while FCS allows to study fast protein exchange.

Yet, such analysis could potentially dramatically change our perception of some of the molecular processes at work in the vicinity of the DNA lesions. A relevant example is the one regarding PARP1 trapping at DNA lesions. As described above, PARP1 recruits rapidly at sites of damage where it triggers the grafting of ADP-ribose chains on nearby targets. The main target of PARylation is PARP1 itself and this process is essential for the dissipation of the protein from DNA lesions, although the precise underlying mechanism remains unclear (Juhász et al., 2020; Prokhorova et al., 2021). The inhibition of the catalytic activity of PARP1 via small-molecule inhibitors leads to a sustained accumulation of this protein at DNA lesions, a process referred to as PARP1 trapping (Murai et al., 2012). A precise definition of this trapping mechanism is essential since it is at the basis of the cytotoxicity of the PARP inhibitors used in the clinic to treat BRCA-deficient tumors. Nevertheless, while chromatin fractionation assays suggest that inhibited PARP1 is stably bound to chromatin (Murai et al., 2012), Fluorescence Recovery After Photobleaching (FRAP) experiments demonstrate that there is a rapid turnover of inhibited PARP1 at DNA lesions (Shao et al., 2020), thus challenging the classical trapping model. This example illustrates the importance of analyzing protein turnover and we will describe below the approaches that are currently available to study this question (Figure 2).

### Population Approaches to Assess Protein Turnover at Sites of Damage

#### Fluorescence Recovery After Photoperturbation

One of the most commonly available techniques to assess protein turnover is FRAP as well as its closely related derivatives based on fluorophore photoactivation or photoconversion instead of photobleaching (Bancaud et al., 2010). In the following, we shall refer to all these methods by using the generic FRAP acronym, in which the last letter refers to photoperturbation instead of photobleaching. The basic principle of FRAP is to locally perturb the steady-state spatial distribution of the fluorescence in a cell expressing a protein of interest tagged with a fluorophore. Analyzing how fluorescence redistributes in space and time after this initial perturbation gives access to the local dynamics of the protein. Therefore, performing FRAP at the sites of DNA damage, which could have been induced by laser irradiation but also other approaches such as nucleases, allows for the assessment of the dynamics of the interaction between repair factors and the DNA lesions (Mortusewicz et al., 2007; Kleppa et al., 2012; Campalans et al., 2013).

A classical way to analyze FRAP recovery curves is to estimate the time needed to recover half of the fluorescence lost upon photobleaching (Bancaud et al., 2010). The visual inspection of the recovery curves may also allow the identification of different populations of molecules differing by the stability of their association with DNA lesions. While this semi-quantitative analysis is useful to compare the behavior of different repair factors or the impact of a given cell treatment on protein turnover (Kimura and Cook, 2001), it does not allow for the assessment of the core components regulating this turnover. To go further, one needs to fit the FRAP recovery curves with appropriate models that include the three parameters that can affect this recovery, the diffusion of the protein, assessed by its diffusion coefficient *D*, as well as its binding and unbinding rates (*k*_*on*_ and *k*_*off*_) to DNA lesions (Sprague et al., 2004). Depending on the relative values of these three parameters, protein dynamics follow different regimes. If the characteristic time spent bound to the lesions is long compared to the time spent to move from one binding sites to the next, the proteins are within a so-called reaction-limited regime (Sprague and McNally, 2005). In the opposite situation, the protein dynamics are considered as diffusion-limited. Then, a mixed regime corresponds to the intermediate scenarios. Defining which regime better describes the behavior of the protein of interest is essential for fitting the FRAP data with the appropriate model (McNally, 2008). Furthermore, depending on the reaction-diffusion regime, it might not be possible to properly estimate the three parameters mentioned above. For example, in the diffusion-limited, only a ratio of *k*_*on*_ and *k*_*off*_ can be estimated (Beaudouin et al., 2006). Noteworthy, while the fitting of the FRAP recovery curves potentially allows for *D* and *k*_*off*_ to be estimated, it does not directly give access to the *k*_*on*_ but rather to a pseudo first-order binding rate *k′_*on*_* that correspond to the product between the actual *k*_*on*_ and the local concentration of binding sites, which could be DNA breaks or other substrate depending on the studied protein.

Therefore, in combination with the analysis of the recruitment kinetics, the FRAP data can provide relevant information about the mechanisms regulating protein accumulation at sites of damage. Estimating the *k*_*on*_ and *k*_off_ rates would allow one to assess whether, for example, the reduced recruitment of a repair protein A upon knock-down of a co-factor B, is due either to a decrease in the *k*_*on*_, meaning defective association at the DNA lesions, or an increase in the *k*_*off*_, that would correspond to an impaired retention within the repair focus (Smith et al., 2019). Distinguishing between these two hypotheses dramatically impacts the interpretation of the role of the co-factor B in the regulation of the accumulation of protein A at sites of damage. Nevertheless, such fitting approach requires good quality data, which is not always achievable due to the limited time resolution of the FRAP assay. To be able to monitor proteins displaying very fast turnover, it is necessary to switch to fluorescence correlation spectroscopy, that will be described in the next section.

#### Fluorescence Correlation Spectroscopy

Fluorescence correlation spectroscopy (FCS) relies on the analysis of fluorescence fluctuations arising from the displacements of fluorescently tagged proteins entering or exiting the parked confocal spot of a laser-scanning setup (Schwille et al., 1997). Focusing the laser beam at the site of damage allow the assessment of protein dynamics within this area (Jeyasekharan et al., 2010; Smith et al., 2019). To quantitatively characterize the dynamics of the proteins, an auto-correlation curve is derived from the fluctuation traces. Similar to half-recovery time derived from the FRAP curves, semi-quantitative estimates such as the characteristic residency time within the focal volume can be obtained from the FCS data. However, based on the same original model as the one used for FRAP, it is also possible to estimate the main parameters controlling protein dynamics at sites of damage, the diffusion coefficient *D* as well as the *k*_*on*_ and *k*_*off*_ rates (Michelman-Ribeiro et al., 2009).

Because FCS characterizes protein dynamics at higher sampling rates than FRAP, it can be used to assess faster turnover and also allow a better characterization of the diffusive component. In particular, it can distinguish pure Brownian motion from an anomalous sub-diffusive behavior that would arise from motion hindering by the high level of crowding in the nuclear space (Bancaud et al., 2009). FCS acquisitions with variable sizes of the probed volume allow the characterization of the diffusional behavior of proteins to be pushed even further (Wawrezinieck et al., 2005; Abdisalaam et al., 2014). In particular, White et al. used such approach to demonstrate that transcription factors explore the nuclear environment by alternating 3D diffusion in the nucleoplasm with transient association with DNA potentially involving 1D diffusive sliding (White et al., 2016). This same approach could be applied to the analysis of DNA repair factors in determining whether they follow the same strategy of nuclear exploration when searching for DNA lesions.

As mentioned above, FCS is more appropriate than FRAP to assess fast protein turnover. Conversely, because it requires that the proteins move within the focal volume to generate signal, FCS is blind to slow turnover. In the following, we will describe a third method that aims at filling the gap in accessible timescales between FCS and FRAP.

#### Analysis by Pair Correlation Functions

Pair correlation function (pCF) is based on the analysis of fluorescence fluctuations measured along a confocal line scan that arise from the movements of individual fluorescently tagged proteins (Digman and Gratton, 2009). The acquisition of fluorescent signal during a line scan brings an added spatial dimension to fluctuation-based analysis compared to the static FCS. During analysis, the fluorescence signals from two different pixels along the acquisition line are cross-correlated, allowing the characteristic transit time of a given molecule between these two pixels to be estimated. Therefore, pCF has the ability to assess protein dynamics slower than those accessible by FCS and still remains faster than FRAP since it only requires scanning a single line. While this technique has been primarily used to describe the movement of proteins across different cells or cellular compartments (Clark et al., 2016; Hinde et al., 2016), it has recently been applied to the characterization of the turnover of the repair factor of 53BP1 at DNA repair foci (Lou et al., 2020). Using a two-color version of pair correlation analysis, the authors showed that 53BP1 binds to the repair foci as dimer but dissociates from these foci as monomer. This first application of pair correlation to the DNA repair field demonstrates that this technique has the potential to bring unique information about the dynamics of repair factors at sites of damage in the future.

### Single-Molecule Approaches to Assess Protein Turnover at Sites of Damage

Recruitment data or fluorescence recovery curves acquired by live cell fluorescence imaging characterize the dynamics of repair proteins at the population level. Fluorescence correlation spectroscopy or pair correlation monitor fluctuations arising from single molecules, but these fluctuations are averaged over time and therefore these methods only give access to a mean behavior of the proteins. Thus, there is a need for an approach to monitor the behavior of repair factors at the single molecule level. Indeed, reaching such resolution would bring invaluable information in particular regarding the way the repair proteins navigate within the nucleus to find the DNA lesions and associate with these lesions. Several super-resolution methods have been proposed to break the diffraction limit and gain access to structural details below ∼150 nm. In the DNA repair field, the gain in spatial resolution brought by these approaches contributed to uncover new functions of proteins participating to the DDR. For example, the characterization of repair foci by super-resolution imaging highlighted the importance of 53BP1 in the maintenance of the local chromatin conformation in the vicinity of the sites of DNA damage (Ochs et al., 2019). Colocalization at the scale of few tens of nanometers helped to prove that BRCA2 contributes to the recruitment of RNASEH2A and control the levels of DNA:RNA hybrids at DSBs (D’Alessandro et al., 2018).

Among these super-resolution methods, single molecule light microscopy (SMLM) has shown to be useful not only in bringing structural insights but also to characterize protein dynamic. When applied in living cells, SMLM gives access to the trajectories of individual proteins (Izeddin et al., 2014). Key features of the initial steps of the DDR can be extracted from the quantitative analysis of these trajectories. Uphoff and coworkers used SMLM to determine the dynamics of polymerase I and ligase molecules searching for DNA gaps and nicks in live *Escherichia coli* cells and estimated that these two factors need about 10 s to find their substrate within the cells and 2 s to resolve it (Uphoff et al., 2013). This finding asks the question of the strategies used by the repair factors to find their target and reconcile the two opposite requirements of an efficient search process: being fast and specific. Multiple *in vitro* data on naked DNA demonstrate that repair proteins perform facilitated diffusion to optimize this search process, alternating between 3D exploration phases and 1D diffusion along the DNA (D’Augustin et al., 2020). Nevertheless, it is unclear whether this also holds true when the DNA is wrapped around nucleosomes and folds in the complex multiscale architecture observed in cell nuclei. Only few publications report 1D diffusion along the DNA in living cells (Hammar et al., 2012; Esadze et al., 2017). Instead, a common feature of multiple nuclear proteins looking for rare targets along the chromatin is their propensity to alternate stretches of 3D diffusion with short unspecific chromatin binding events (Reuter et al., 2014; Normanno et al., 2015; Jones et al., 2017). Whether these transient associations with chromatin underly 1D diffusion along the DNA or not remains unclear but theoretical models indicate that a fine regulation of the switch between DNA-bound and diffusive phases is essential to ensure a rapid finding of the target (Bénichou et al., 2011).

Besides the analysis of these search mechanisms, SMLM approaches also allow the behaviour of the repair proteins to be followed at later stages of the DDR in order to investigate how the local environment established nearby the DNA lesions influences the dynamic behavior of the repair factors. In a recent report, Miné-Hattab et al. analyzed the individual trajectories of Rad52 (the functional analog of human BRCA2) and RFA1 (a member of the RPA complex) in *Saccharomyces cerevisiae* (Miné-Hattab et al., 2021). They demonstrate that RFA1 displays subdiffusive motions similar to those reported for the break itself, while Rad52 shows Brownian motion within the repair foci. Therefore, while both factors accumulate at the repair foci, this accumulation is triggered by two different mechanisms: RFA1 binds strongly to DSBs, in agreement with its role in the protection of single-stranded DNA (Wold, 1997), in contrast to Rad52, which could be confined within the repair foci due to liquid-liquid phase separation mechanisms (Oshidari et al., 2020). Such detailed analysis comes from the unique ability of SMLM to analyze the trajectories of single molecules, demonstrating its high potential to address complex questions in the DNA repair field.

## The Need for a Quantitative Model to Integrate the Data Obtained From Different Tools

### The Motivations for the Use of Mathematical Models to Analyze the Spatio-Temporal Dynamics of Repair Factors

Mathematical and computational models are increasingly used to help investigate biological systems in relation to a wide variety of experimental data. In cell biology, the focus has extensively been on cell signaling pathways (Aldridge et al., 2006), leading to the creation of hundreds of models, from a couple of interacting components to huge networks comprising many interacting molecules (Luijsterburg et al., 2010). The objective is to build a model based on reasonable assumptions regarding the behavior of the proteins at scales that are not readily accessible experimentally and to use this model to generate simulated outputs, such as recruitment curves at sites of DNA damage, that could be fitted to the real data (Lengert and Drossel, 2013; Tobias et al., 2013). This allows for the validation of whether the chosen model adequately catches the complexity of the studied biological process and, if so, for the estimation of the values of the unknown parameters of the model. Obviously, the better the initial knowledge about the process, the easier it is to build-up a meaningful model and then fit it to the data to get a precise estimation of the few remaining unknowns.

### The Basic Principles Governing the Establishment of Reaction-Diffusion Models

**FIGURE 3:**
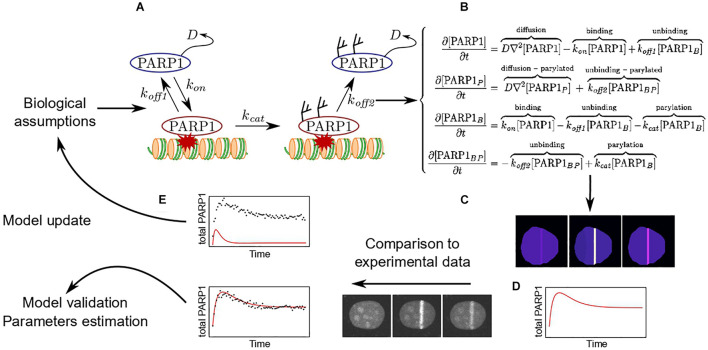
Development of a mathematical model to assess PARP1 dynamics at DNA lesions. **(A)** Based on specific biological assumptions, it is possible to build a model describing the different states of PARP1 in and out the DNA damage area as well as the exchange rates between these different states. **(B)** The model can be transcribed in a set of reaction-diffusion differential equations. **(C)** Solving the set of equations allows to predict the evolution of the spatio-temporal distribution of PARP1 within the nucleus in response to induction of DNA damage. **(D)** Based on this simulation, it is possible to obtain predicted recruitment kinetic curves. **(E)** These simulated recruitment curves can be fitted to the experimental ones. If the model is unable to fit the experimental data, a new model needs to be rebuilt. In contrast, if the model allows to properly fit the data, it is possible to estimate the parameters included in the model.

To be more illustrative and explain how to build a model describing the behavior of repair factors at sites of damage, we shall take the specific example of PARP1. As discussed previously, PARP1 can be either in a PARylated or an unPARylated form and is either bound to the lesions, where it is catalytically active, or diffuse within the nucleus. Based on this description, one can build the simple model presented on Figure 3. This model is a simplified representation of reality and the conclusion that we will be able to draw when fitting it with the experimental data will be limited to the assumptions we made to build it. Here, for example, a critical assumption is that PARylated PARP1 cannot bind to DNA lesions (Figure 3A).

Once the elementary components of the model are established, its mathematical transcription can take two alternative forms: deterministic and stochastic (Cowan et al., 2012). Deterministic models are able to predict the spatio-temporal evolutions of concentrations of proteins according to a set of differential equations describing all the diffusion-reaction processes that impact these concentrations (Figure 3B). The stochastic models focus on the molecular scale. The state of each molecule is simulated in space and time and the choice between different elementary events (displacement due to diffusion, binding, etc.), is defined randomly at each time step based on probabilities derived from the reaction rates. By construction, these two types of models have different applications. While deterministic models are used to fit experimental outputs encompassing large number of molecules (recruitment curves, FRAP recovery), which mean behavior is estimated at the population level by their local concentration, the stochastic approach can catch the random characteristics associated with small number of molecules assessed by methods such as FCS or single molecule tracking (Axelrod et al., 1976; Furlan et al., 2019). Both deterministic and stochastic models can then be solved numerically using dedicated software such as Copasi, Virtual Cell, or Berkeley Madonna (Klipp et al., 2005; Cowan et al., 2012), allowing for the prediction of the spatio-temporal evolution of the protein distribution within the whole nucleus upon induction of DNA lesions (Figure 3C). Ultimately, it is then possible to generate outputs simulating those obtained experimentally (Figure 3D).

### Integrating Data From Different Sources to Improve Model Predictions

Fitting the simulated outputs to the experimental data allows a first estimation of whether the model that was chosen is suitable for the studied process. Following the parsimony principle, one should start with the simplest possible model and progressively add extra components only when the simple model does not accurately represent the experimental data (Tyson and Novák, 2015). For example, in the context of the process described in Figure 3, one could build up model that is even simpler by assuming that the PARylated status of PARP1 does not impact its ability to interact with chromatin. Nevertheless, in such simplified situation, the simulated recruitment curves would only display an accumulation phase but no dissipation, showing that some key components of the biological process are missing in the model. Several quantitative tools based on the parsimony principle are available to guide the choice between two models of differential complexities, such as the Akaike or the Bayesian information criteria (Burnham and Anderson, 2002). Combined with biological knowledge about the studied process, they allow the definition of models that properly describe the experimental data with the least number of parameters.

Nevertheless, parsimony does not necessarily imply unicity, meaning that two models of similar complexity may equally fit the same experimental outputs. To choose between these two models, it is often necessary to provide additional knowledge to the system by including data from other assays or analyzing the response of the biological system to different perturbations. An interesting example in the context of protein recruitment to DNA lesions, is the report by Lengert et al. (2015). They performed FRAP experiments at the sites of damage at different timings of the recruitment kinetics. By adjusting these combined FRAP/recruitment data with different models that were equally fitting pure recruitment curves, they show that they are able to discriminate the most suitable model. Including additional information from other assays may also reduce the number of unknown parameters, thus facilitating the estimation of the remaining ones. The example model shown on Figure 3 is composed of 5 unknown parameters. However, the diffusion coefficient of unbound PARP1 can be easily estimated by FCS or FRAP experiment in undamaged nuclei. Similarly, the binding and unbinding rates for the unPARylated PARP1 could probably be estimated by assessing the turnover of a catalytically inactive mutant of PARP1 at sites of damage, using FRAP or FCS. Therefore, ultimately, it would be possible to reduce the number of unknown parameters to only 2.

In summary, we showed in this section how mathematical models could be used to better interpret complex dynamics of repair proteins at DNA lesions. Plugging different experimental data into the model, assessing not only the local concentration of the repair factors at sites of damage but also its turnover, will help to establish complex robust models allowing to improve our understanding of the multiple steps of the DDR.

## Conclusion

With the increasing number of quantitative live-cell microscopy techniques used within labs specialized in DNA repair, there comes the promise of future insights in the field due to the possibilities offered by imaging multiple aspects of protein dynamics. In particular, while protein recruitment curves at sites of laser irradiation have been analyzed extensively within the last years, only few reports have exploited methods such as FRAP, FCS, or pair correlation, to characterize the turnover of repair proteins at DNA lesions. Yet, this information is critical to better understand how these proteins interact with their substrate and accumulate within the repair foci. Furthermore, the recent progresses in SMLM methods for tracking the motions of single proteins within the nucleus now open a new avenue to investigate some aspects of the DDR that, so far, could only be addressed *in vitro* on naked DNA or reconstituted chromatin. This is particularly the case of the fascinating question of the search mechanisms employed by the initial repair factors to ensure the efficient detection of their target within the dense and highly complex nuclear space, a key event that is at the basis of the initiation of the whole DDR.

## Author Contributions

All authors contributed to the design, review, and proofreading of the manuscript. SZ and MJ prepared the figures.

## Conflict of Interest

The authors declare that the research was conducted in the absence of any commercial or financial relationships that could be construed as a potential conflict of interest.

## Publisher’s Note

All claims expressed in this article are solely those of the authors and do not necessarily represent those of their affiliated organizations, or those of the publisher, the editors and the reviewers. Any product that may be evaluated in this article, or claim that may be made by its manufacturer, is not guaranteed or endorsed by the publisher.
